# Radio-Frequency Characteristics of Stacked Metal–Insulator–Metal Capacitors in Radio-Frequency CMOS Devices

**DOI:** 10.3390/mi17010054

**Published:** 2025-12-30

**Authors:** Tae Min Choi, Hwa Rim Lee, Sung Gyu Pyo

**Affiliations:** School of Integrative Engineering, Chung-Ang University, Seoul 156-756, Republic of Korea

**Keywords:** stacked MIM, RFIC, CMOS, capacitance

## Abstract

This paper describes the radio-frequency (RF) characteristics of stacked metal–insulator–metal (MIM) capacitors used in RF CMOS technology. To ensure accurate analysis, various de-embedding methods for stacked MIM capacitors were verified, and an improved RF model was constructed accordingly. To develop an equivalent circuit for the improved RF model by analyzing the RF characteristics of stacked MIM capacitors, we compared de-embedding methods for measured stacked MIM capacitors: one-step (open-pattern or short-pattern) de-embedding and two-step (combined open-pattern and short-pattern) de-embedding. For the analysis of stacked MIM capacitors, at least two-step de-embedding was used, while for precise de-embedding, three-step de-embedding using a thru pattern was employed. Based on the measured values obtained using these two-step de-embedding methods, a modified equivalent circuit was constructed. This circuit was analyzed based on various parameters, including MIM capacitance, quality factor, S-parameter, and Y-parameter, and the results were comparatively examined. The findings highlight outstanding accuracy of the modified model, which is maintained even in high frequency bands.

## 1. Introduction

With the rapid development of wireless communication technology, radio-frequency (RF) resources have become indispensable, with materials, components, and circuits operating in the RF band being in high demand. These RF components and circuits are continuously being researched as the basis for integrated circuits (ICs) that can achieve not only high performance but also miniaturization, weight reduction, and cost-effectiveness [1,2,3,4,5,6]. Specifically, Si-based RFICs are circuits that integrate active and passive components and utilize CMOS-based active components and passive components, such as resistors, inductors, and capacitors, according to the characteristics of the circuit. The characteristics of each component directly impact the overall performance of such circuits; hence, improving the characteristics of both active and passive components is crucial [7,8,9,10,11,12]. Capacitors, a representative passive component, are crucial in RFICs, serving various purposes, including impedance matching, DC blocking in bias circuits, bypass circuits for preventing low-frequency oscillation and improving noise characteristics, and degeneration circuits for increased stability and linearity [13,14,15,16,17].

However, metal–insulator–metal (MIM) capacitors, along with inductors, have the disadvantage of occupying a large area. Research is being conducted in two main areas to overcome this limitation. First, increasing the capacitance density involves changing the type or thickness of the insulator [18,19]. Second, various structural design methods for MIM capacitors can be employed to increase the capacitance density per unit area [20,21,22]. The MIM capacitor used in this study is a stacked-type unit, the structural design of which involves vertically connecting two MIM capacitors in parallel to increase the capacitance density per unit area.

Based on their RF characteristics, typical capacitors do not function as pure capacitors. This is because as the frequency increases, the capacitor is affected by parasitic components, such as parasitic inductance and parasitic resistance, in addition to the original capacitance. Ideally, the impedance of a capacitor should decrease linearly with increasing frequency. However, as the frequency increases, the impedance increases significantly due to the influence of parasitic inductance and parasitic resistance [23,24,25]. Recent studies have extensively explored RF modeling techniques for conventional MIM capacitors, with efforts focused on accurately capturing frequency-dependent degradation caused by parasitic inductance, resistance, and substrate-related loss mechanisms [17,26,27,28,29]. Advanced modeling approaches have incorporated multi-layer dielectric coupling, extended substrate networks, and frequency-dependent parasitic extraction to improve prediction accuracy in the multi-GHz regime [30,31,32]. However, despite these developments, research on the RF modeling of stacked MIM capacitors remains limited. Stacked architectures introduce additional parasitic paths associated with vertically interconnected electrodes and inter-metal dielectrics, which are not sufficiently addressed in traditional single-layer MIM capacitor models. Due to the increasing demand for high-density capacitors in RF CMOS applications, establishing an accurate RF model that reflects the unique parasitic behavior of stacked MIM structures has become a critical requirement. This study aims to introduce an improved RF model. In contrast to earlier stacked MIM capacitor models, which primarily represented two MIM units connected in parallel with simplified parasitic branches [11], the present work adopted a refined topology that independently accounts for electrode-dependent parasitic paths and the additional series impedance introduced by the bottom electrode. Furthermore, the proposed model employs a rigorous parameter-extraction procedure based on two-step open–short de-embedded S-parameters and validates the extracted elements against the full frequency dependence of the Y-parameter, yielding substantially improved agreement compared to conventional approaches. Unlike previous models typically verified up to approximately 10 GHz, the proposed model demonstrates consistent accuracy from 500 MHz to 20 GHz, highlighting its suitability for high-frequency RF CMOS applications requiring reliable stacked-capacitor behavior across an extended GHz range.

## 2. Experimental Procedure

### 2.1. Fabrication of Stacked MIM Capacitors

**Figure 1:**
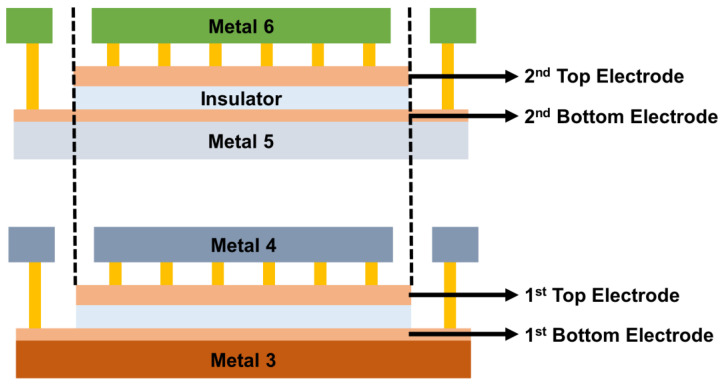
Plan view and cross-sectional view of stacked MIM capacitor.

The MIM capacitors used in the study were manufactured using a 0.18-μm standard CMOS process. The MIM stacked structure with two vertically parallel connected structures corresponded to the stacked structure of TiN/SiN_x_/TiN capacitor single units previously implemented in research results [11]. The stacked MIM capacitor module used in the analysis was structured to achieve twice the capacitance per unit area by connecting the first MIM capacitor implemented on Metal 3 and the second MIM capacitor formed on Metal 5 in parallel. The test pattern of the stacked MIM capacitor was laid out to evaluate electrical characteristics such as capacitance density, leakage current, VCC, and TCC. The unit sizes of the first and second MIM capacitors included in each module were 10 μm × 10 μm, 15 μm × 15 μm, 20 μm × 20 μm, 25 μm × 25 μm, 30 μm × 30 μm, and 50 μm × 50 μm. To determine the RF characteristics of the stacked MIM capacitor alone, without any effect of the degree of mismatch for the capacitor, the test pattern of the capacitor in the unit form (excluding the array form) was used in the analysis. Figure 1 briefly illustrates the cross-sectional structure and design rule of the stacked MIM capacitor.

### 2.2. RF Measurement Setup and Calibration

The structure of the ground signal from the ground pad was utilized to assess the wafer of the test pattern using a microwave vector network analyzer (8510C, Agilent Technologies, Santa Clara, CA, USA).

Measurements for RF analysis were performed at high frequencies, ranging from several gigahertz to several tens of gigahertz. In such cases, conventional I–V and C–V methods cannot be used, and S-parameter measurements are required instead. S-parameter values measured using a network analyzer can vary significantly depending on the level of calibration, which necessitates accurate calibration. The RF measurement environment for stacked MIM capacitors is summarized below:

1. Measurement range: 500 MHz to 20 GHz;
2. Measurement equipment: HP8510C Vector Network Analyzer (Agilent Technology);
3. RF probe: Cascade Infinity GSG Probe;
4. Calibration method: Two-port short, open, load, and thru calibration.

### 2.3. De-Embedding Procedure

**Figure 2:**
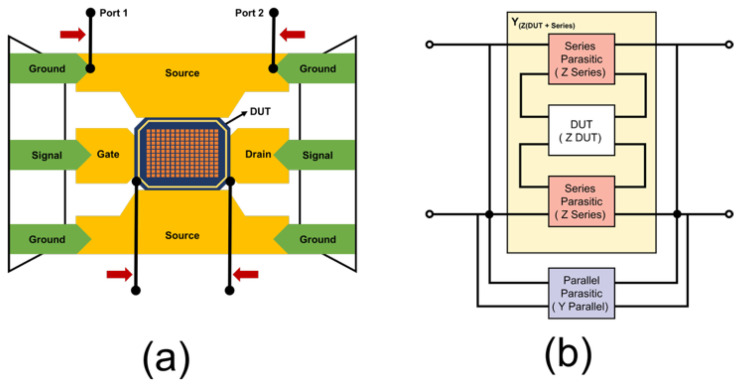
Test patterns with (**a**) extra pattern and (**b**) parasitic elements.

Accurate RF characterization of stacked MIM capacitors requires the removal of parasitic contributions originating from probe pads, interconnects, and measurement fixtures. To extract the intrinsic RF response of the device under test (DUT), de-embedding was performed using open and short calibration structures fabricated alongside the DUT. The measurement test pattern and the associated parasitic elements introduced by the pad and interconnect structures are illustrated in Figure 2. In Figure 2a, Ports 1 and 2 denote the signal terminals of the two-port VNA measurement and are applied to the signal pads of the DUT. The adjacent pads correspond to the ground (G) terminals of the GSG probe and provide the RF return path. The underlying metal interconnect routing is not left floating; it electrically connects the signal pads to the DUT electrodes and is included in the signal path. The two metal lines beneath the DUT correspond to the interconnect routing between the probe pads and the DUT, which introduce additional parasitic elements that must be removed through the de-embedding procedure. Although the simplified illustration may visually suggest a connection to the ground pads, the actual electrical excitation follows the conventional G–S–G contact scheme.

**Figure 3:**
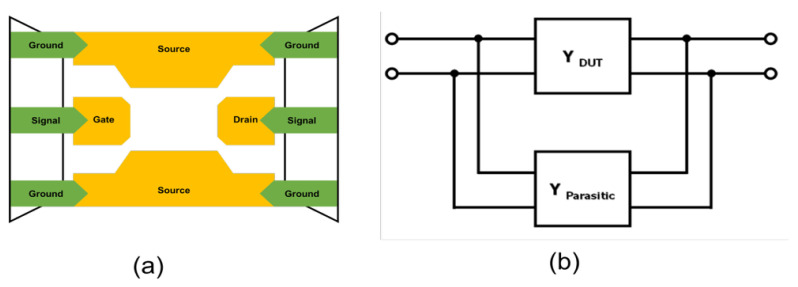
Open de-embedding patterns with (**a**) extra pattern and (**b**) parasitic elements.

Open de-embedding was first applied to eliminate parallel parasitic components associated with pad and interconnect capacitances. As illustrated in Figure 3, the open de-embedding structure represents these parasitic elements in the admittance domain. At this stage, the physical configuration remains identical to that shown in Figure 3a,b, where the same GSG probe connection and access interconnect routing are assumed, and only the termination condition at the DUT location differs between the short and open structures. The measured S-parameters were converted into Y-parameters, and the parasitic admittance extracted from the open pattern was subtracted from the total admittance, as expressed in Equation (1).

Y_DUT(Open)_ = Y_Total_ − Y_Open_. (1)

**Figure 4:**
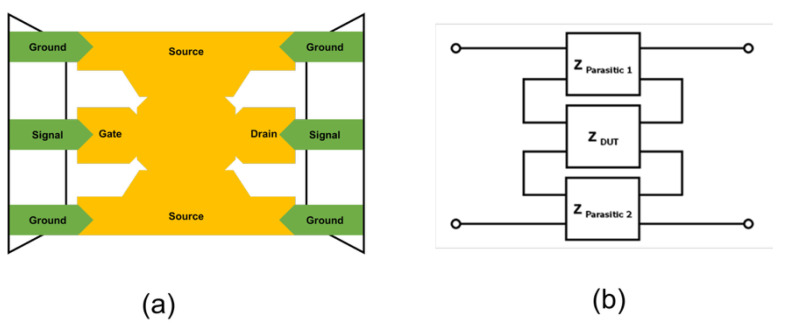
Short de-embedding patterns with (**a**) extra pattern and (**b**) parasitic elements.

Subsequently, short de-embedding was employed to remove series parasitic components introduced by metal interconnects and probe access paths. The short de-embedding structure, shown in Figure 4, expresses these parasitic elements in the impedance domain. In Figure 4a, the metal structure represents the interconnect path that electrically links the probe pad to the DUT. This metal path provides the current flow into the stacked MIM capacitor and introduces series parasitic resistance and inductance associated with the access routing. Therefore, the short de-embedding pattern is used to characterize and remove these series parasitic components, enabling the accurate extraction of the intrinsic RF response of the DUT. The open-de-embedded Y-parameters were converted into Z-parameters, and the impedance contribution obtained from the short pattern was subtracted, as described by Equations (2)–(7). Accordingly, Y_(short-open)_ represents the combined admittance of the pad capacitance and access interconnect parasitics defined in the equivalent circuit of Figure 3a, with no additional structural elements introduced.

Z_DUT(Short)_ = Z_Total −_ Z_Short_. (2)

Y_DUT(Open)_ = Y_Total_ − Y_Open_, (3)

Y_(Short)(Open)_ = Y_Short_ − Y_Open_, (4)

Z_DUT(Open)_ = Z_(YDUT(Open))_ (Y-parameter Z-parameter conversion), (5)

Z_Short(Open)_ = Z_(YShort(Open))_, (6)

Z_DUT_ = Z_DUT(Open)_ − Z_Short(Open)_, (7)

Equation (7) is derived under the same physical and electrical configuration as Equations (4)–(6), and therefore shares the identical GSG probe connection and interconnect topology illustrated in Figure 4a,b. This equation does not correspond to a new schematic or connection diagram; rather, it represents a subsequent algebraic de-embedding step that removes the previously identified parasitic admittance to extract the intrinsic DUT response.

Finally, the de-embedded Z-parameters were converted back into S-parameters to obtain the intrinsic RF characteristics of the stacked MIM capacitors, as summarized in Equation (8). This two-step open–short de-embedding procedure enables reliable extraction of capacitance, quality factor, and Y-parameter characteristics over a wide frequency range.

S_DUT_ = S_(ZDUT)_ (Z-parameter S-parameter conversion). (8)

In this study, the two-step de-embedding approach was adopted because the pad dimensions and interconnect lengths of the test structures are electrically short within the measurement frequency range. Under these conditions, transmission-line effects are negligible, and the additional complexity associated with three-step de-embedding using a thru structure provides limited benefit.

## 3. Results and Discussion

The de-embedding method removes parallel and serial parasitic components by using both open and short de-embedding patterns. Open de-embedding is performed using the Y-parameter of the test pattern, while short de-embedding is performed using the Z-parameter of the open de-embedded short pattern; the de-embedded patterns are then converted into S-parameters to obtain the true S-parameters of the DUT. RF analysis is performed using the S-parameters thus extracted. Equations (3)–(8) summarize the process of open and short pattern de-embedding.

### 3.1. RF Characteristics Analysis Based on De-Embedding Method

**Figure 5:**
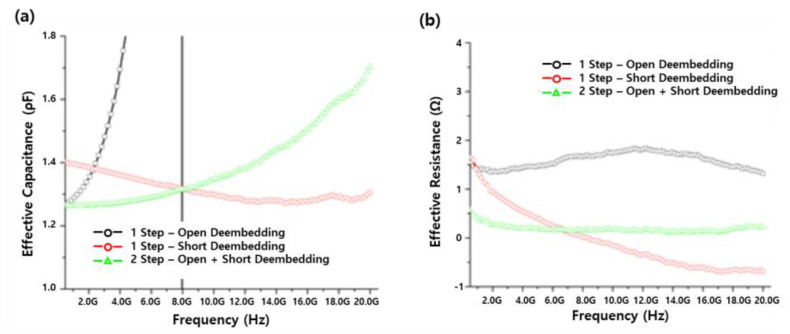
Effective (**a**) capacitance and (**b**) resistance with frequencies for stacked MIM module, obtained using different de-embedding methods.

Precise RF measurements are essential for developing the equivalent circuit for an accurate RF model. To address discrepancies in the effective capacitance values extracted from measured stacked MIM capacitors depending on the de-embedding method, one-step (open-pattern or short-pattern) de-embedding and two-step (combined open-pattern and short-pattern) de-embedding were compared and analyzed. The open de-embedding pattern resulted in under-de-embedding due to residual parasitic components in the area where the DUT was formed. In contrast, the short de-embedding pattern resulted in over-de-embedding as it de-embedded the entire area where the DUT was formed. Using both open and short de-embedding yielded reliable data. Figure 5a displays the effective capacitance of the unit stacked MIM capacitor of the stacked module among the Al-based stacked MIM capacitors. In the case of under de-embedding open de-embedding, the total effective capacitance was significantly larger than that of fine de-embedding open, short de-embedding, and in the case of over de-embedding short de-embedding, the total effective capacitance was smaller than that of fine de-embedding open, short de-embedding. Figure 5b shows the parasitic total effective resistance of the module. In the future, at least two-step de-embedding, i.e., open + short de-embedding, should be used for analyzing stacked MIM capacitors, while three-step de-embedding using thru patterns should be employed for precise de-embedding. The three-step de-embedding method is required when the interconnect routing between the probe pads and the DUT becomes electrically long at high frequencies or when substantial pad-to-pad coupling occurs, making the simple two-step open–short removal insufficient to isolate the intrinsic device response. In such cases, the thru structure is used to extract and remove the transmission-line characteristics of the interconnects, thereby enabling accurate modeling of phase delay and frequency-dependent series impedance. In contrast, the present work employed a two-step method because the pad pitch, metal routing length, and operating frequency range place the interconnects well within the electrically short regime, where transmission-line effects are negligible. Under these conditions, the accuracy improvement gained by switching to three-step de-embedding is typically modest—on the order of 1–2% in extracted capacitance and below 0.5 dB in S-parameter magnitude at the upper frequency limit—while the added complexity does not yield proportional benefit. Therefore, the two-step open–short de-embedding scheme was appropriately adopted here, while emphasizing that the three-step method becomes necessary only when interconnect-induced phase errors or line impedance are no longer negligible.

### 3.2. RF Modeling Based on Analysis of RF Characteristics of Stacked MIM Capacitors

For a conventional MIM capacitor, RF behavior is typically represented by a model in which the intrinsic capacitance is connected in series with parasitic inductance and resistance associated with the electrode structure, while parasitic capacitance and substrate-related resistance are connected in parallel to account for inter-metal dielectric coupling and substrate loss. This widely adopted model has been shown to effectively describe the frequency-dependent degradation induced by parasitic components [11,33]. Parasitic components extracted using the general equivalent circuit are summarized in Table 1.

However, the stacked MIM capacitors examined in this study differs in that two dielectric capacitor units are vertically connected in parallel. As demonstrated in our previous work [11], each capacitor unit contributes an individual intrinsic capacitance and possesses its own parasitic inductance and resistance components due to the corresponding electrode path. Therefore, when the two MIM capacitor layers are stacked, the parasitic components associated with the top and middle conductive layers must be considered independently, while the bottom electrode introduces an additional series parasitic path. Furthermore, the parasitic coupling between the stacked structure and the inter-metal dielectric/substrate was previously modeled using parallel parasitic capacitance and resistance. To evaluate the validity of the proposed RF model, the measured RF characteristics of the stacked MIM capacitor reported in previous work [11] were used as the reference dataset.

**Figure 6:**
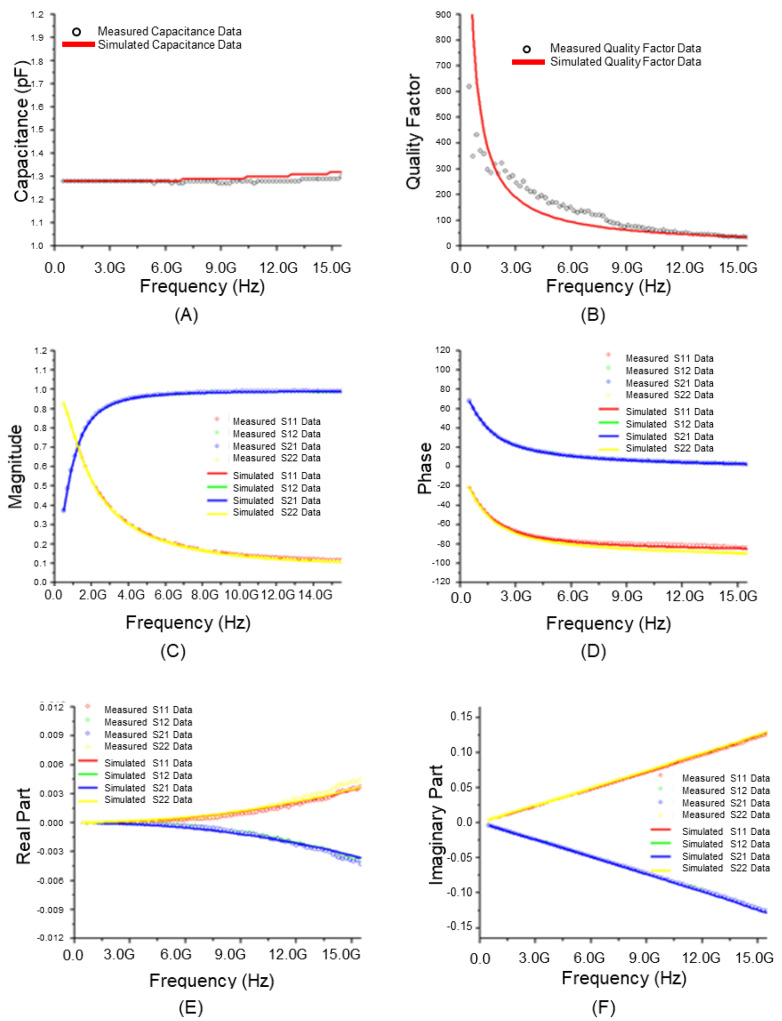
Characteristics of conventional RF model of stacked module (25 μm × 25 μm): (**A**) Total effective capacitance; (**B**) Quality factor; (**C**) Magnitude of S-parameter; (**D**) Phase of S-parameter; (**E**) Real part of Y-parameter; (**F**) Imaginary part of Y-parameter.

**Figure 7:**
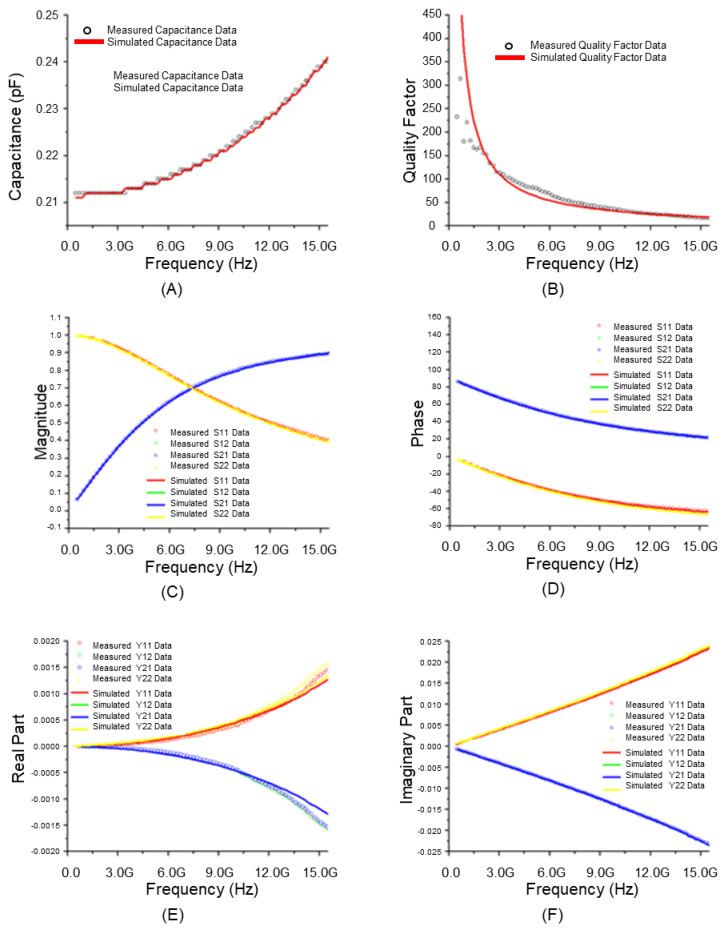
Characteristics of modified RF model of stacked module (10 μm × 10 μm): (**A**) Total effective capacitance; (**B**) Quality factor; (**C**) Magnitude of S-parameter; (**D**) Phase of S-parameter; (**E**) Real part of Y-parameter; (**F**) Imaginary part of Y-parameter.

Figure 6 presents the results of analyzing the RF model accuracy of a stacked MIM capacitor for each parameter using the equivalent circuit of a general RF model. The equivalent circuit of a typical MIM capacitor RF model includes the MIM capacitance (C_MIM_), which acts as a direct capacitor, along with a parasitic inductance (L_s_) and resistance (R_s_) component occurring in series with this capacitor. Additionally, it was configured with a combination of parasitic capacitance (C_ox_, C_Sub_) and parasitic resistance (R_Sub_) components occurring in parallel with these series components. Figure 7 shows the corresponding results based on the equivalent circuit of the improved RF model. Figure 7A highlights the degree of agreement with the actual total effective capacitance (Cmim1 + Cmim2) as the sum of the formed MIM Capacitances 1 and 2. The measured value and the extracted simulated value were almost identical. Thus, the accuracy was higher when the equivalent circuit of the general RF model (Figure 7A) was used. The accuracy remained high up to high frequencies. Figure 7B shows high accuracy as the quality factor, which is an important parameter for evaluating the characteristics of stacked MIM capacitors. Figure 7C–F compares the magnitude and phase values of the S-parameter, which can be used to evaluate the accuracy of the model, based on the real and imaginary parts of the Y-parameter, respectively. Excellent accuracy of the model is underscored by the fact that the measured value and the extracted simulated value were almost identical up to a high frequency band. For the Y-parameter, which is more important than the S-parameter in the analysis of capacitor characteristics, a significantly more accurate result was obtained when the equivalent circuit of the improved RF model was used with respect to RF model was used. Based on the analysis of the extracted RF model of the stacked module in terms of the MIM capacitance, quality factor, S-parameter, and Y-parameter, excellent accuracy could be observed, given that the measured values and the extracted simulated values were almost identical up to a high frequency band. Considering these results, stacked MIM capacitors can perform stably in higher frequency bands if a ground shield is included to reduce degradation at high frequencies or if the RF isolation of each stacked MIM capacitor is increased to reduce parasitic capacitance.

## 4. Conclusions

In this study, we sought to identify the problems associated with stacked MIM capacitors and suggest potential solutions by analyzing the RF characteristics of such capacitors. Moreover, we constructed an improved RF model for a stacked MIM capacitor and obtained accurate simulation data accordingly. The RF analysis was performed using integration processing and an Al-based stacked MIM capacitor. To create an equivalent circuit of an accurate RF model, one-step (open-pattern or short-pattern) de-embedding and two-step (combined open-pattern and short-pattern) de-embedding were compared when analyzing the measured stacked MIM capacitor. The findings suggest that at least two-step de-embedding should be used for the analysis of stacked MIM capacitors and that three-step de-embedding using thru patterns should be employed for precise de-embedding. A modified equivalent circuit was constructed using the measured values obtained via two-step de-embedding. The modified equivalent circuit constructed in this study was analyzed in terms of various parameters, such as MIM capacitance, quality factor, S-parameter, and Y-parameter, and the results were comparatively examined. The findings underscore the excellent accuracy of the modified model, which was sustained up to a high frequency band. The results for the modified equivalent circuit indicate that additional research is needed to manufacture stacked MIM capacitors to reduce degradation at high frequencies, which can be achieved by inserting a ground shield, or to reduce parasitic capacitance, which is possible by increasing the RF isolation of each stacked MIM capacitor. These techniques could allow stacked MIM capacitors to perform stably in higher frequency bands.

## Figures and Tables

**Table 1:** RF model parameters of typical RF model MIM capacitors.

MIM (μm^2^)	10 × 10	15 × 15	20 × 20	25 × 25	30 × 30
L_S_ (pH)	19.04	11.74	6.26	2.61	0.78
R_S_ (mΩ)	2.28	0.76	0.35	0.22	0.18
C_MIM_ (fF)	216.00	466.40	825.90	1277.00	1782.00
C_ox_ (fF)	4.71	7.59	11.11	1.25	20.02
C_Sub_ (fF)	2902.00	1370.88	836.10	589.08	455.17
R_Sub_ (Ω)	7.16	18.77	35.32	56.83	83.30

## Data Availability

The original contributions presented in this study are included in the article. Further inquiries can be directed to the corresponding author.

## References

[1] Levinger R., Shumaker E., Banin R., Ravi A., Degani O. (2022). The rise of the digital rfic era: An overview of past and present digital rfic advancements. IEEE Microw. Mag..

[2] Robertson I.D., Lucyszyn S. (2001). RFIC and MMIC Design and Technology.

[3] Ellinger F., Claus M., Schröter M., Carta C. Review of advanced and beyond cmos fet technologies for radio frequency circuit design. Proceedings of the 2011 SBMO/IEEE MTT-S International Microwave and Optoelectronics Conference (IMOC 2011).

[4] Malm B.G., Haralson E., Johansson T., Ostling M. (2005). Self-heating effects in a bicmos on soi technology for rfic applications. IEEE Trans. Electron Devices.

[5] Sia C.B., Ong B.H., Yeo K.S., Ma J.-G., Do M.A. (2005). Accurate and scalable rf interconnect model for silicon-based rfic applications. IEEE Trans. Microw. Theory Tech..

[6] Yang R., Qian H., Li J., Xu Q., Hai C., Han Z. (2006). Soi technology for radio-frequency integrated-circuit applications. IEEE Trans. Electron Devices.

[7] Mukhopadhyay R., Park Y., Sen P., Srirattana N., Lee J., Lee C.-H., Nuttinck S., Joseph A., Cressler J.D., Laskar J. (2005). Reconfigurable rfics in si-based technologies for a compact intelligent rf front-end. IEEE Trans. Microw. Theory Tech..

[8] Bennett H.S., Brederlow R., Costa J.C., Cottrell P.E., Huang W.M., Immorlica A.A., Mueller J.-E., Racanelli M., Shichijo H., Weitzel C.E. (2005). Device and technology evolution for si-based rf integrated circuits. IEEE Trans. Electron Devices.

[9] Watson A., Mayevskiy Y., Francis P., Hwang K., Srinivasan G., Weisshaar A. Compact modeling of differential spiral inductors in si-based rfics. Proceedings of the 2004 IEEE MTT-S International Microwave Symposium Digest (IEEE Cat. No. 04CH37535).

[10] Sia C.B., Ong B.H., Lim W.M., Yeo K.S., Alam T. (2008). Modeling and layout optimization of differential inductors for silicon-based rfic applications. IEEE Trans. Electron Devices.

[11] Sul W.S., Pyo S.G. (2014). Rf characteristic analysis model extraction on the stacked metal–insulator–metal capacitors for radio frequency applications. IEEE Trans. Electron Devices.

[12] Sul W.S., Kwon S.H., Choi E., Cui Y., Lee K.W., Shim H.J., Gao Y., Hahn S.J., Pyo S.G. (2017). Radiofrequency characteristics of ionized sputtered tantalum nitride thin-film resistor in cmos device. Electron. Mater. Lett..

[13] Mindan B., Hong L. The analysis of impedance matching problem in rf circuit design. Proceedings of the 2010 International Forum on Information Technology and Applications.

[14] Chongcheawchamnan M., Karacaoglu U., Robertson I.D. (2005). Radio-frequency integrated circuits. Encyclopedia of RF and Microwave Engineering.

[15] Chen C., Huang D., Zhao Y., Jin Y., Yang J. (2023). An ultra-low-voltage 2.4-ghz flicker-noise-free rf receiver front end based on switched-capacitor hybrid tia with 4.5-db nf and 11.5-dbm oip3. IEEE J. Solid-State Circuits.

[16] Molinero D., Aghaei S., Morris A.S., Cunningham S. (2019). Linearity and rf power handling on capacitive rf mems switches. IEEE Trans. Microw. Theory Tech..

[17] Kannadassan D., Sivasankaran K., Kumaravel S., Cheng C.-H., Baghini M.S., Mallick P. (2024). High-*k* metal–insulator–metal capacitors for rf and mixed-signal vlsi circuits: Challenges and opportunities. Proc. IEEE.

[18] Choi T.-M., Jung E.-S., Yoo J.-U., Lee H.-R., Yoon S., Pyo S.-G. (2025). I–v characteristics and electrical reliability of metal–sixny–metal capacitors as a function of nitrogen bonding composition. Micromachines.

[19] Choi T.M., Jung E.S., Yoo J.U., Lee H.R., Pyo S.G. (2024). Capacitance-voltage fluctuation of si(x)n(y)-based metal-insulator-metal capacitor due to silane surface treatment. Micromachines.

[20] Yu X., Zhu C., Hu H., Chin A., Li M.F., Cho B.J., Kwong D.-L., Foo P.D., Yu M.B. (2003). A high-density mim capacitor (13 ff/μm2) using ald hfo2 dielectrics. IEEE Electron Device Lett..

[21] Klootwijk J.H., Jinesh K.B., Dekkers W., Verhoeven J.F., Heuvel F.C.v.d., Kim H.D., Blin D., Verheijen M.A., Weemaes R.G.R., Kaiser M. (2008). Ultrahigh capacitance density for multiple ald-grown mim capacitor stacks in 3-d silicon. IEEE Electron Device Lett..

[22] Guo Y., Wang S., Du X., Liang S., Huang S., Peng S., Xie Y., Ma M., Xiong L. (2023). Construction of ultrahigh capacity density carbon nanotube based mim capacitor. Energy Storage Mater..

[23] Gutiérrez-Vicente V., Torres-Torres J.A., Torres-Torres R. (2024). Broadband s-parameter-based characterization of multilayer ceramic capacitors submitted to mechanical stress through bending tests on a pcb. Micromachines.

[24] Ye Y., Cheng K.W.E. (2017). Analysis and optimization of switched capacitor power conversion circuits with parasitic resistances and inductances. IEEE Trans. Power Electron..

[25] Gurov E.V., Uvaysov S.U., Uvaysova A.S., Ivanov I.A. Analysis of the parasitic parameters influence on the analog filters frequency response. Proceedings of the 2019 International Seminar on Electron Devices Design and Production (SED).

[26] Aniktar H., Savcı H.Ş. (2023). Numerical and measurement based modeling of a mim capacitor in a 0.25 µm sige-c bicmos process. Prog. Electromagn. Res. C.

[27] Wang L., Xu R.-M., Yan B. (2006). Mim capacitor simple scalable model determination for mmic application on gaas. Prog. Electromagn. Res..

[28] Mu J., Chou X., Ma Z., He J., Xiong J. (2018). High-performance mim capacitors for a secondary power supply application. Micromachines.

[29] Moon H., Yu S., Song S.S., Nam I. (2009). Characterization and modeling of stacked mim on-chip capacitors with high-capacitance density up to 20 ghz frequency region. Microw. Opt. Technol. Lett..

[30] Cai W.Z., Shastri S.C., Azam M., Hoggatt C., Loechelt G.H., Grivna G.M., Wen Y., Dow S. Development and extraction of high-frequency spice models for metal-insulator-metal capacitors. Proceedings of the 2004 International Conference on Microelectronic Test Structures (IEEE Cat. No.04CH37516).

[31] Tseng V.F.G., Xie H. (2014). Increased multilayer fabrication and rf characterization of a high-density stacked mim capacitor based on selective etching. IEEE Trans. Electron Devices.

[32] Piquet J., Cueto O., Charlet F., Thomas M., Bermond C., Farcy A., Torres J., Fléchet B. Simulation and characterization of high-frequency performances of advanced mim capacitors. Proceedings of the 35th European Solid-State Device Research Conference, 2005. ESSDERC 2005.

[33] Liao E.B., Li H., Guo L.H., Lo G.Q., Kumar R., Balasubramanian N., Kwong D.L. (2007). Rf, dc, and reliability performance of mim capacitors embedded in organic substrates by wafer-transfer technology (wtt) for system-on-package applications. IEEE Trans. Electron Devices.

